# Contrast phase recognition in liver computer tomography using deep learning

**DOI:** 10.1038/s41598-022-24485-y

**Published:** 2022-11-24

**Authors:** Bruno Aragão Rocha, Lorena Carneiro Ferreira, Luis Gustavo Rocha Vianna, Luma Gallacio Gomes Ferreira, Ana Claudia Martins Ciconelle, Alex Da Silva Noronha, João Martins Cortez Filho, Lucas Salume Lima Nogueira, Jean Michel Rocha Sampaio Leite, Maurício Ricardo Moreira da Silva Filho, Claudia da Costa Leite, Marcelo de Maria Felix, Marco Antônio Gutierrez, Cesar Higa Nomura, Giovanni Guido Cerri, Flair José Carrilho, Suzane Kioko Ono

**Affiliations:** 1 InRad, Institute of Radiology, University of São Paulo, School of Medicine, Rua Dr. Ovídio Pires de Campos, 75 Cerqueira César, São Paulo SP, 05403-010 Brazil; 2 Machiron Ltd., Rua Capote Valente, 671, São Paulo, 05409-002 Brazil; 3 Informatics Department, The Heart Institute, Hospital das Clínicas (HCFMUSP), University of São Paulo, School of Medicine, Rua Dr. Enéas de Carvalho Aguiar 44, São Paulo, SP 05403-000 Brazil; 4 Department of Gastroenterology, University of São Paulo, School of Medicine (FMUSP), Hospital das Clínicas (HCFMUSP), Rua Dr. Enéas Carvalho de Aguiar, 225, São Paulo, SP 05403-000 Brazil

**Keywords:** Hepatology, Machine learning, Computed tomography

## Abstract

Hepatocellular carcinoma (HCC) has become the 4th leading cause of cancer-related deaths, with high social, economical and health implications. Imaging techniques such as multiphase computed tomography (CT) have been successfully used for diagnosis of liver tumors such as HCC in a feasible and accurate way and its interpretation relies mainly on comparing the appearance of the lesions in the different contrast phases of the exam. Recently, some researchers have been dedicated to the development of tools based on machine learning (ML) algorithms, especially by deep learning techniques, to improve the diagnosis of liver lesions in imaging exams. However, the lack of standardization in the naming of the CT contrast phases in the DICOM metadata is a problem for real-life deployment of machine learning tools. Therefore, it is important to correctly identify the exam phase based only on the image and not on the exam metadata, which is unreliable. Motivated by this problem, we successfully created an annotation platform and implemented a convolutional neural network (CNN) to automatically identify the CT scan phases in the HCFMUSP database in the city of São Paulo, Brazil. We improved this algorithm with hyperparameter tuning and evaluated it with cross validation methods. Comparing its predictions with the radiologists annotation, it achieved an accuracy of 94.6%, 98% and 100% in the testing dataset for the slice, volume and exam evaluation, respectively.

## Introduction

Over the past few years, diagnostic medicine has achieved prominent success in the development of tools based on machine learning (ML) algorithms, especially by deep learning techniques such as convolutional neural networks (CNN), which are the most used and suitable approach for imaging analysis^1^. This computer-oriented approach enables the extraction of imaging patterns based on the ability to learn from data and has been successfully applied in diagnosis and treatment of several conditions, including melanoma, nail mycosis, pneumonia, acute respiratory distress syndrome (ARDS) and coronavirus disease, as well as in liver diseases, such as hepatocellular carcinoma (HCC)^2–7^.

Hepatocellular carcinoma has become the 4th leading cause of cancer-related deaths, with an increasing incidence, especially in western nations^8–10^. Magnetic resonance imaging (MRI) and multiphase computed tomography (CT) are currently the gold standard imaging method for detecting HCC with no need for a biopsy if a typical imaging pattern is present i.e. whenever a mass measuring 1 cm is found that demonstrates arterial hyper-enhancement and one or more major features in selected patients with high risk for HCC, according to the Liver Imaging Reporting and Data System^8,11–14^. These techniques involve intravenous contrast injection with a four-phase image acquisition protocol (unenhanced, arterial, portal and delayed), which is the clinical reference standard.

Nonetheless, liver segmentation and HCC identification pipelines present many challenges related to the input images quality. For instance, Dercle et al.^15^ demonstrated that the quality of CT scans acquired at the portal venous phase was suboptimal in one third of colorectal cancer patients. In addition, although radiologists can easily recognize the study phase visually, automatic phase identification is important for the deployment of future HCC screening algorithms. In the best scenario, automatic identification of CT scan phases should not be a problem, since there is a field called SeriesDescription in the DICOM metadata^16^, which has the name of the acquired series. However, in a real-life and clinical setting, the main problem is that there is no standard for naming across machines, even on machines of the same manufacturer. The series acquisition time DICOM Tag could also be a way to identify the series phase. However, this approach would only work if all exams had all four phases in clinical practices. As many exams may have fewer phases (for example, only the unenhanced and portal phase), this reinforces the importance of identifying the phase directly by analyzing the image.

The lack of standardization and proper quality control of contrast-enhanced phases in abdominal CT scans is a pivotal limitation in the field of radiomics^17^, and, to the best of our knowledge, very few attempts have been made to address these issues^18^.

Hence, these problems motivated this group to implement an algorithm to automatically classify the CT scans phases in Hospital das Clínicas (HCFMUSP) database in São Paulo, Brazil. We created the Liver Artificial Intelligence (HepatIA) platform to store the CT scans and facilitate the radiologists annotation of the exams. Using an unprocessed DICOM folder containing all four contrast phases as input, we implemented a fully-automated CNN algorithm that outputs a sorted folder for each of the relevant contrast phases.

### Organization of article

The introduction of this article describes our motivation to identify the contrast phases in a CT scan. In the following section, there is an overview of our methodology and a description of HepatIA platform. In “Methods” section, we present a detailed description of data collected, annotation process, algorithm construction and evaluation methods. Then, we present the results, discussion and conclusion. After the references, we added the acknowledgements, author contributions statement, additional information about the dataset and competing interests.

## Overview of the methodology

**Figure 1:**
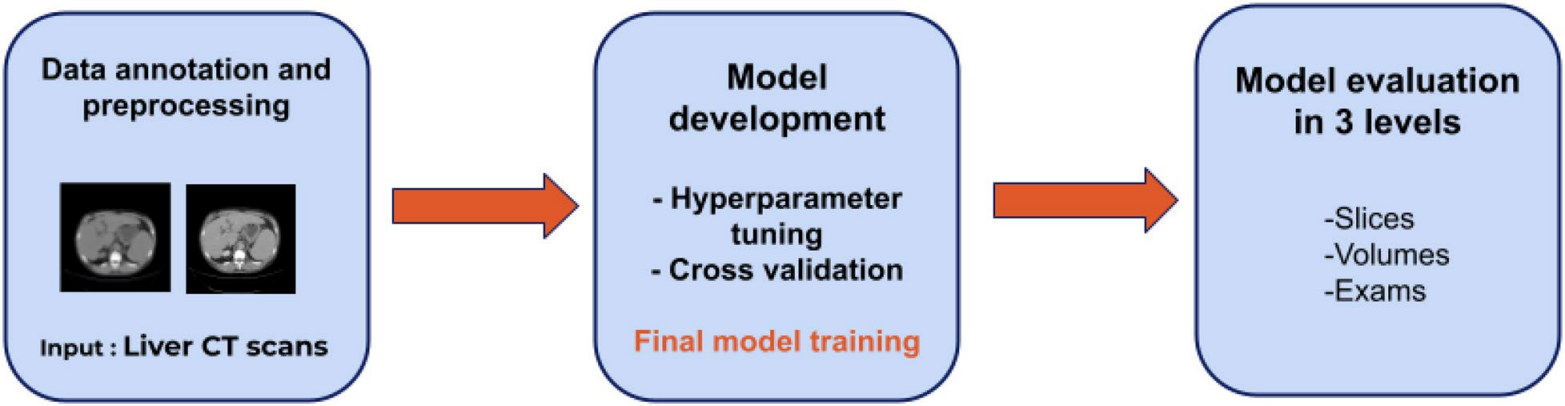
Overview of the methodology and application. Our methodology is divided into three main steps: (1) data annotation and preprocessing, (2) model development and (3) model evaluation.

As shown in Fig. 1, the data used in this paper are abdominal CT scans acquired with a protocol for evaluation of the liver, which can be evaluated in three levels: (1) slice, which is a single image of the exam; (2) volume, which is the group of slices that belongs to a single contrast phase; and (3) exam, which includes the four volumes from the corresponding four phases. The training step was performed in two steps. Firstly, we performed a hyperparameter tuning to find the best combination of parameters for the convolution neural network (CNN). Secondly, we trained the defined model with our dataset. For a better evaluation of the final structure of our model, we included a cross validation step. The developed CNN analyzes slices, generating the probability of the slice belonging to each phase. For the analysis of volumes and exams, we combined the results from the slices with a post-processing technique. More details of the dataset and methods used is described in “Methods” section.

## Methods

### Study population

The study was approved by Ethics Committee at the *Hospital das Clinicas da Faculdade de Medicina da Universidade de São Paulo* under the study protocol CAAE 69385217.1.0000.0068 in accordance with the ethical guidelines of the 1975 Declaration of Helsinki. The need for written informed consent was formally waived by Ethics Committee at the *Hospital das Clínicas da Faculdade de Medicina da Universidade de São Paulo* due to its retrospective, single-center nature. Our study population is composed of 396 CT scans from unique patients, which are composed with 4 volumes each, summing in 178.633 slices. The data are from healthy liver donors (20%) and cirrhotic patients (80%), from the Division of Clinical Gastroenterology and Hepatology of the Hospital das Clínicas at the University of São Paulo (HCFMUSP) in São Paulo, SP, Brazil, from 2008 to 2021. These patients underwent abdominal Multi-phase Contrast-enhanced Computed Tomography (CT) to assess liver conditions using a 4-phase protocol (unenhanced, arterial, portal-venous and delayed).

### Database and preprocessing

**Figure 2:**
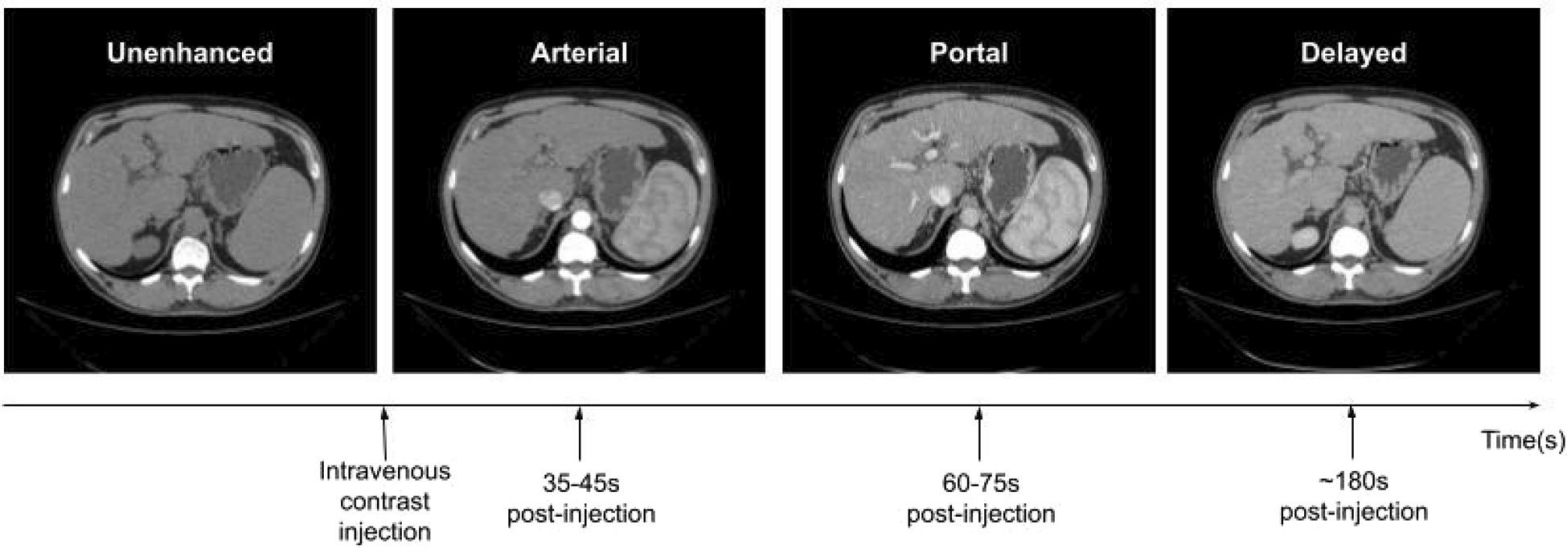
Demonstration of the contrast phases of a liver CT scan.

As our exams are obtained with a four-phase image acquisition protocol, the exam’s volumes should be labeled indicating the phase, which are: unenhanced phase (also known as non-enhanced) is an image acquisition before the administration of intravenous contrast; Arterial phase is an acquisition about 35–45 s after intravenous contrast injection; portal phase is 60–75 s post-injection and delayed phase is about 3 minutes post-injection (Fig. 2).

For the creation of the database and dataset preparation, we created a web-based platform called HepatIA implemented with Django 3.2.9, where the CT scans were stored in an Orthanc DICOM server (version 1.5.8) which is connected to the hospital PACS, while patient and clinical-related information was stored in a PostgreSQL relational database^19^.

**Figure 3:**
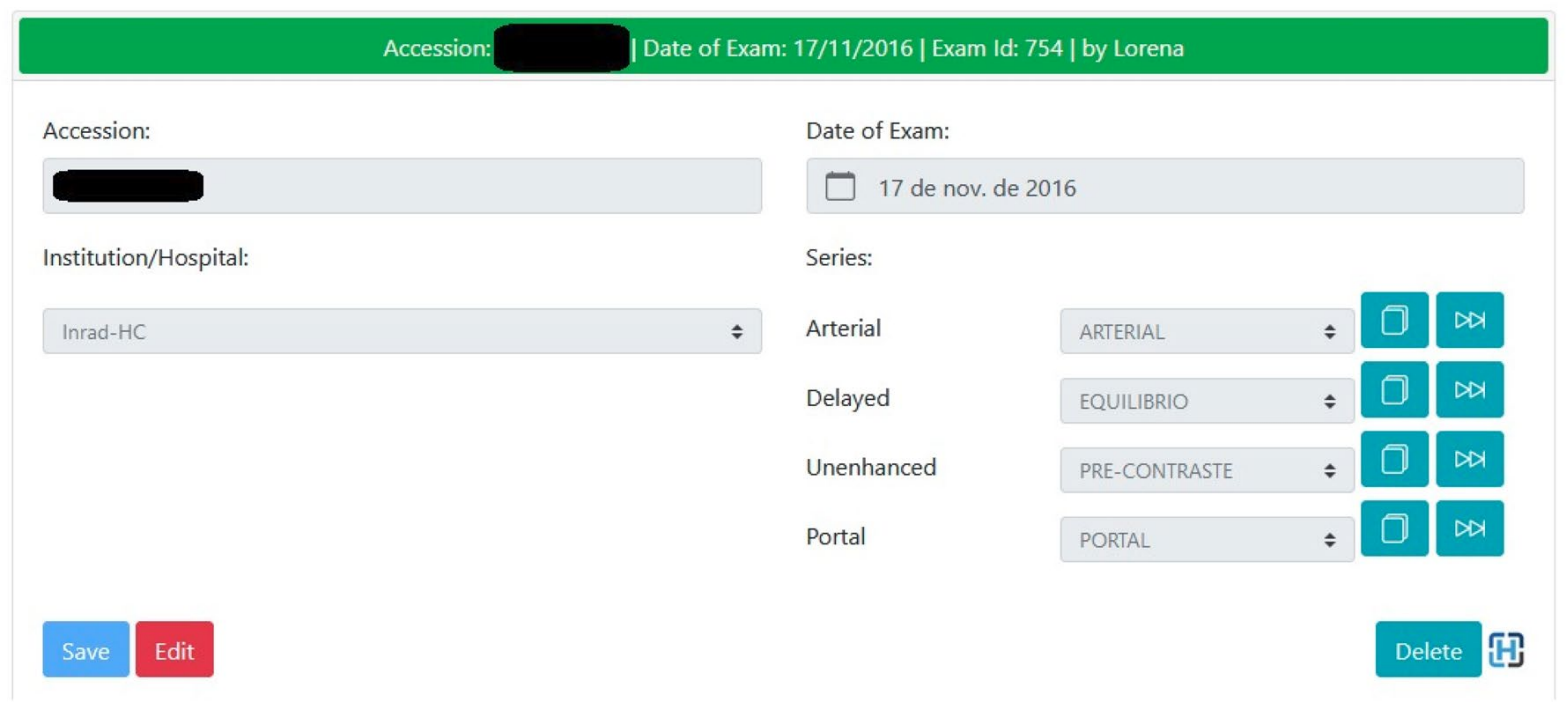
HepatIA platform liver tomography contrast phase annotation screen.

Our platform allows the radiologists to access and fill out exam information, including the correct labels for each of the four contrast phases. This phase annotation was performed by three radiologists with two, four and eleven years of experience, by subjective analysis of the images using the DICOM viewer tool integrated into the HepatIA web-based platform (Fig. 3). Each reader checked the written phase DICOM tag and looked directly at the image to confirm it. We considered this activity to have a very low probability of human error, therefore, we dispensed the need for double reading.

A total of 396 exams (CT scans), from unique patients, were collected in DICOM format. The scans were randomly split into training (80%) and testing (20%). For each CT scan, we used all the four phases and, for each phase, we selected up to 150 slices that were spaced approximately evenly. The exams were taken by different CT machines, of which 214, 91, 90 and 2 are from Philips, GE Medical Systems, Siemens Health and Toshiba, respectively.

### Proposed model

**Figure 4:**
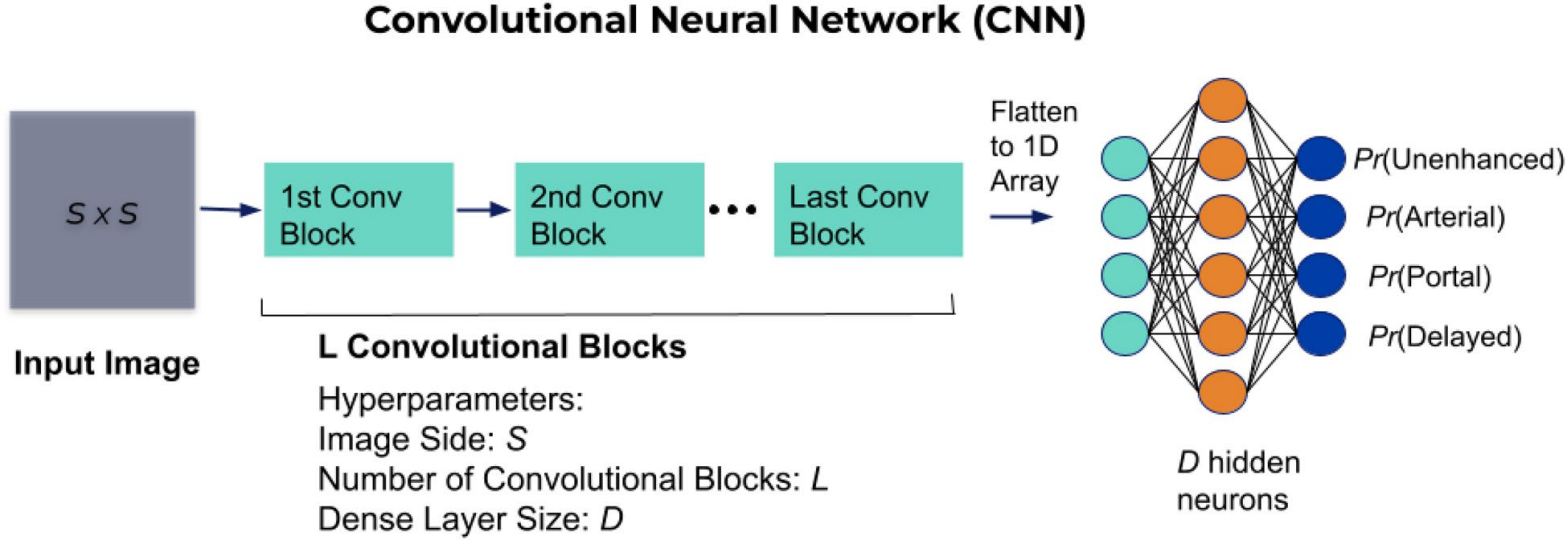
High level description of the CNN architecture.

The implemented model was a Convolutional Neural Network (CNN)^20^ with a dense final classification layer. Figure 4 shows the high level description of the architecture which consists of a series of convolutional blocks with an additional densely connected hidden layer and one output for each of the four contrast phases.

**Figure 5:**
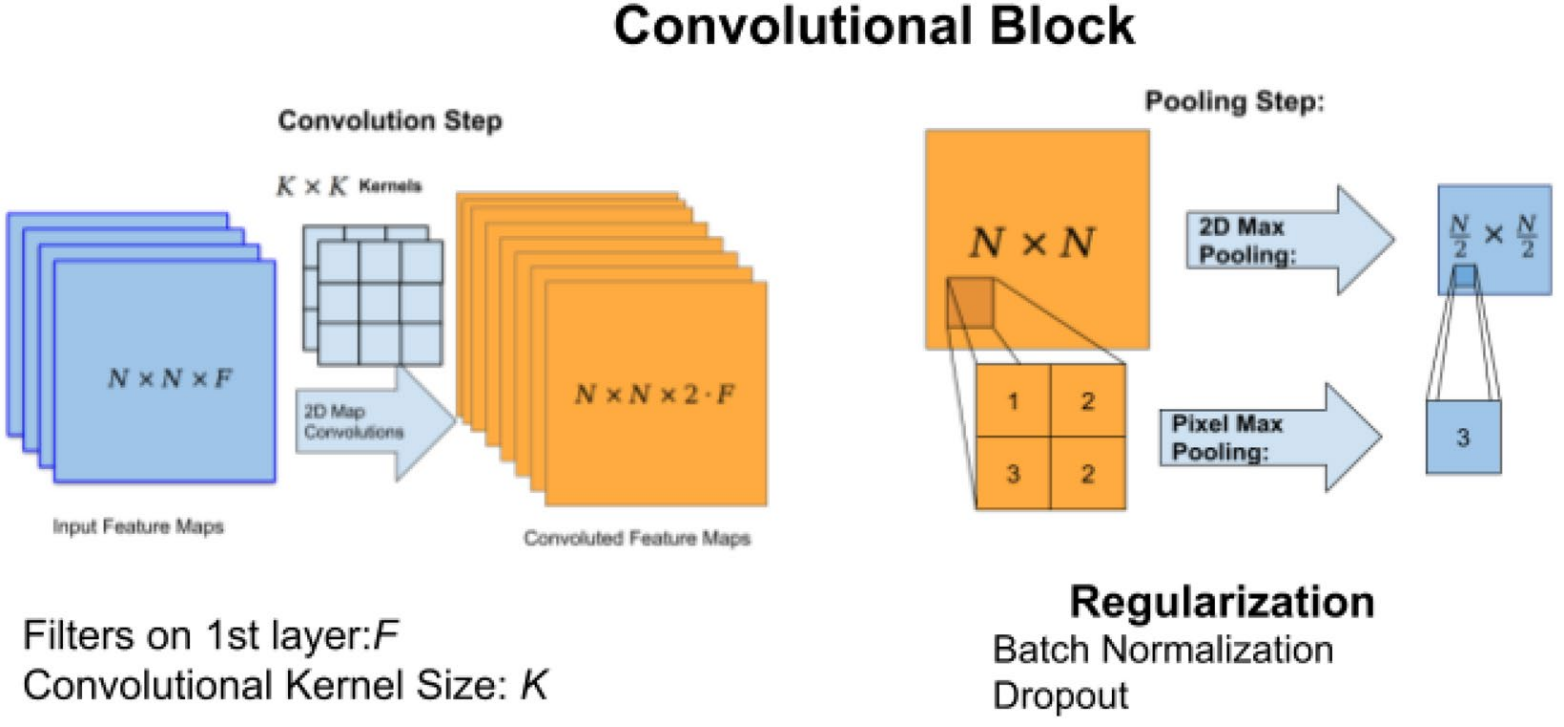
Convolutional block with the convolutional and pooling steps, followed by regularization. *N* input feature size.

The convolutional blocks that we used are detailed on Fig. 5. Each block consists of a convolutional step, which aims to identify patterns in the images, and a pooling step for reducing the image dimensions, and, finally, a regularization step, where batch normalization and dropout are applied.

#### Hyperparameter tuning

The definition of the model depends on several parameters, such as number of convolutional layers, and finding the best combination is not a trivial task. As a solution, we used the Hyperband parameter tuning algorithm^21^ to test the hyperparameters described in Table 1 for all possible values indicated on the table.

**Table 1:** Hyperparameters of the CNN.

Hyperparameter		
Class	Name	Possible values
Architecture	Convolutional layers	2, 3, 4, 5, 6
Architecture	Convolutional kernel	3 $\times$ 3, 5 $\times$ 5, 7 $\times$ 7
Architecture	Filters on 1st layer	8, 16
Architecture	Dense layer size	8, 16, 24, 32
Architecture	Image side	256, 512
Regularizer	Dropout	[1e−6, 1e−2]
Regularizer	Augmentation	Yes or no
Regularizer	Batch normalization	Yes or no
Optimizer	Optimizer	Adam, SGD
Optimizer	Learning rate	[1e−6, 1e−2]

The Hyperband algorithm was chosen since it can test a wide range of hyperparameter sets with few epochs and only does full time training on the most promising combinations, thus allowing many more values to be tested within a reasonable tuning time.

In the case of Dropout and Learning Rate, the tuning algorithm samples values according to a log-based probability distribution, assigning equal probabilities to each order of the magnitude range. The Learning Epochs and Batch values were set after testing for impacts on execution time and memory consumption.

For this step, we used the train data set split into training (80%) and validation (20%). The evaluation of the model with the chosen hyperparameters was made with the test data set. The final parameters of the model are presented in the Results section.

#### Cross-validation

Using the data set that was previously assigned for training purposes and the model defined with the tuning procedure, we performed a *k*-fold cross-validation, where *k* corresponds to both the number of subsets the data was randomly sorted into, as well as the number of iterations. In this investigation, we set *k* as 5. For each iteration, four of the folds were used for training the CNN while the remaining one was used for testing, so that, at the end, each input data (either slices, volumes or exams) was used exactly four times for training and once for testing, respectively.

#### Loss function

In order to measure and minimize the errors over the epochs during the training and validation steps, we used the Categorical Cross-Entropy loss function, defined as:

1 $$\begin{aligned} Categorical\_Cross\_Entropy(o, y, p) = -\sum _{c=1}^M y_{o,c}\log (p_{o,c}) \end{aligned}$$

where $y_{o,c}$ corresponds to a binary indicator, i.e. 1 if class label *c* is the correct classification for this observation and 0 otherwise. *M* corresponds to the classes, which are the four exam phases; Observation *o* is the slice; $p_{o,c}$ is the model predicted probability that observation *o* is from class *c*.

### Evaluation

#### Prediction levels

The algorithm generates predictions in three evaluation levels: individual slices, individual phase volumes and exams. The model analyzes the 3D input images by processing one 2D slice at a time. The algorithm assigns a score to each input slice for all possible phases. Hence, volume-level prediction is made by choosing the corresponding phase with the highest mean score across all slices. Finally, the full exam prediction is made by combining the predictions of its corresponding volumes. Since there is exactly one volume for each phase, in cases where more than one volume has the same volume-level prediction the volume with highest confidence is chosen and the other volume’s prediction is changed to the next available option.

For both slice and volume levels several classification performance metrics are described in the next section. Exam level predictions were evaluated according to the number of volumes correctly identified.

#### Evaluation metrics

Herein, we evaluated the performance of our model in the testing set using the most common metrics for classification problems: F1 score, Area under the ROC curve, accuracy, precision and recall^22^. These metrics were calculated in a one-vs-rest manner .i.e. there will be four measures for each evaluation metric, each of them corresponding to correctly classifying the images as a given class against all the other classes. The means were also reported for each evaluation metric.

The accuracy of the model is calculated as the fraction of images classified correctly over the total number of images:

2 $$\begin{aligned} Accuracy = \frac{TP + TN}{TP + TN + FP + FN} \end{aligned}$$

where *TP*, *FP*, *TN* and *FN* correspond to the number of true positives, false positives, true negatives and false negatives, respectively.

The following metrics are defined based on a class *C*, in this case, the classes are the four phases of the CT scan. The precision is defined as follows:

3 $$\begin{aligned} Precision(C) = \frac{TP(C)}{TP(C) + FP(C)} \end{aligned}$$

while the recall (also referred to sensitivity or true positive rate) corresponds to:

4 $$\begin{aligned} Recall(C) = \frac{TP(C)}{TP(C) + FN(C)} \end{aligned}$$

The formula for the F1 score is defined as:

5 $$\begin{aligned} F1 score (C) = \frac{2 \times {Precision(C)} \times {Recall(C)}}{Precision(C) + Recall(C)} \end{aligned}$$

As the last metric, AUC is the area under the ROC curve, which is the plot of FPR x recall. FPR is given by:

6 $$\begin{aligned} False\_positive\_rate (C) = \frac{FP(C)}{FP(C)+TN(C)} \end{aligned}$$

Each of these metrics is calculated per slice prediction as well as per volume prediction.

Lastly, the performance of the analysis per exam was expressed in terms of how many phases in each exam were correctly classified. In this step, we iteratively associate volumes with phases. The first assignment of a volume-phase pair is done according to the highest prediction among all possible volume-phase pairings. After that, the next highest prediction value for a pairing that does not contain the already paired volume and phase is identified and the corresponding volume and phase are paired. This step will happen until only one volume and one phase remain to be paired. Thus, in the end, an exam will always have each of its 4 volumes associated with a different phase as well as an indicated volume for each of the four phases.

The implementation was performed using Python 3.8 environment with Tensorflow 2.6 and Keras 2.6 packages. The computer used had the following specifications: Linux Ubuntu 20.04 Virtual Machine in a FOXCONN M100-NHI High Processor Computing (HPC) with 32 Cores CPU @ 2.9 GHz 346 GB RAM and a cluster of 16 NVIDIA Tesla V100 16 GB cards.

## Results

### Hyperparameter tuning

Table 2 shows best values for the hyperparameters adjusted with hyperparameter tuning. These values were then used in all further analysis.

**Table 2:** Best hyperparameters after tuning.

Hyperparameter		
Class	Name	Value
Architecture	Convolutional layers	5
Architecture	Convolutional kernel	5 $\times$ 5
Architecture	Filters on 1st layer	32
Architecture	Dense layer size	32
Architecture	Image side	256
Regularizer	L2 *	0.01
Regularizer	Dropout	$2.045 \times 10^{-4}$
Regularizer	Augmentation	No
Regularizer	Batch normalization	Yes
Optimizer	Optimizer	Adam
Optimizer	Learning rate	1.483 $10^{-3}$
Optimizer	Loss function *	Cross entropy
Execution	Learning epochs *	50
Execution	Batch *	5

*Only one value was tested for this hyperparameter.

Comparing a model trained with the default hyperparameter values with a model trained with the best tuned values, we observed an accuracy increase from 78.02 to 85.49% in the slices evaluation and 89.90 to 92.31% in the volume one.

### Best model results

**Figure 6:**
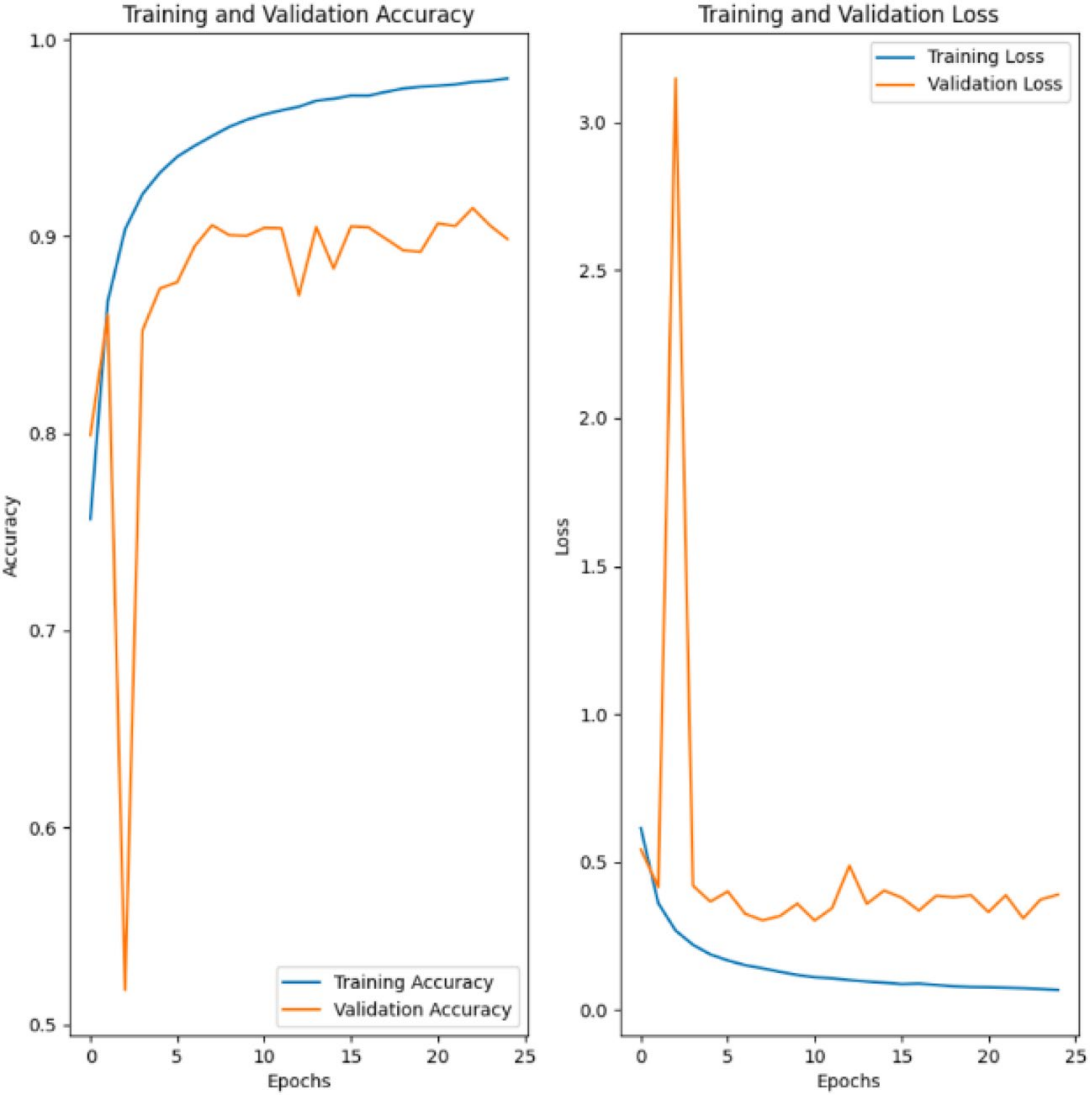
Accuracy and loss in the training and validation data sets of a CNN to identify the four phases of abdominal CT scans.

Figure 6 displays the accuracy and the loss of the model for both training and validation data sets over 25 epochs, that is, 25 rounds of evaluation with all training images. The highest accuracy with the smallest loss was achieved in the 23rd epoch in the validation data set.

**Figure 7:**
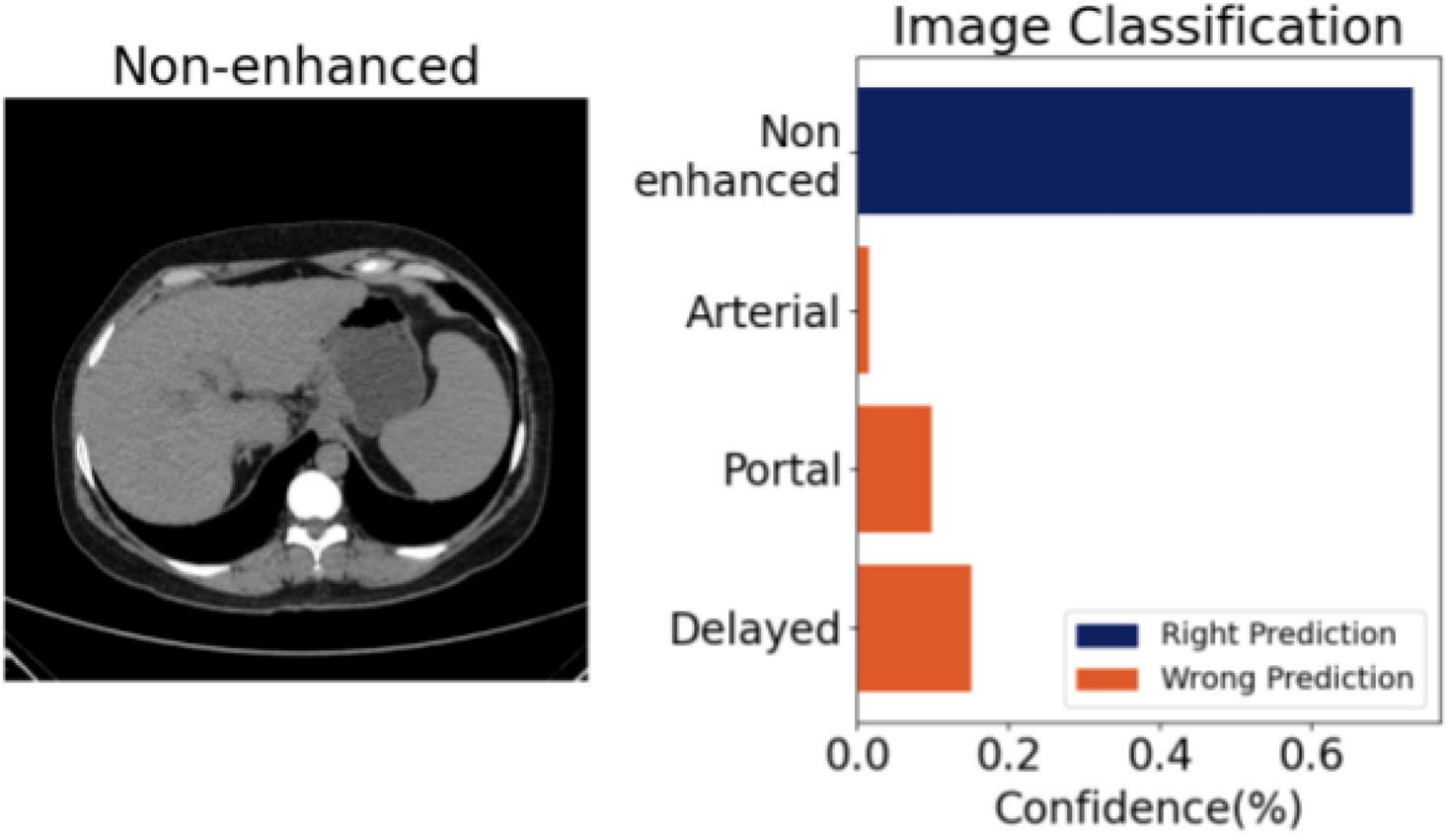
An example of slice with correct prediction of contrast phase. This plot shows the confidence of the slice belonging to each contrast phase. For this slice, the correct phase is *non-enhanced* and the algorithm classified it correctly with 70% of confidence, while it also gave a probability of approximately 18% of belonging to delayed, 10% of belonging to portal and 2% of belonging to arterial.

The output of the model is the probabilities of each slice belonging to each contrast phase, as shown in Fig. 7. The final classification of the slice is the contrast phase with higher probability. The accuracy achieved in the testing set was 94.6%. Among the other metrics, as shown in Table 3, the unenhanced phase had the best results.

**Table 3:** Performance metrics in the testing set for the slices evaluation using the best model found by the Hyperparameter tuning.

	Precision	Recall	F1-score	AUC	N
Unenhanced	0.99	0.99	0.99	1	10,757
Arterial	0.98	0.98	0.98	1	10,870
Portal	0.92	0.89	0.89	0.99	11,907
Delayed	0.89	0.93	0.91	0.99	10,803
Average	0.95	0.95	0.95	0.99	–

**Figure 8:**
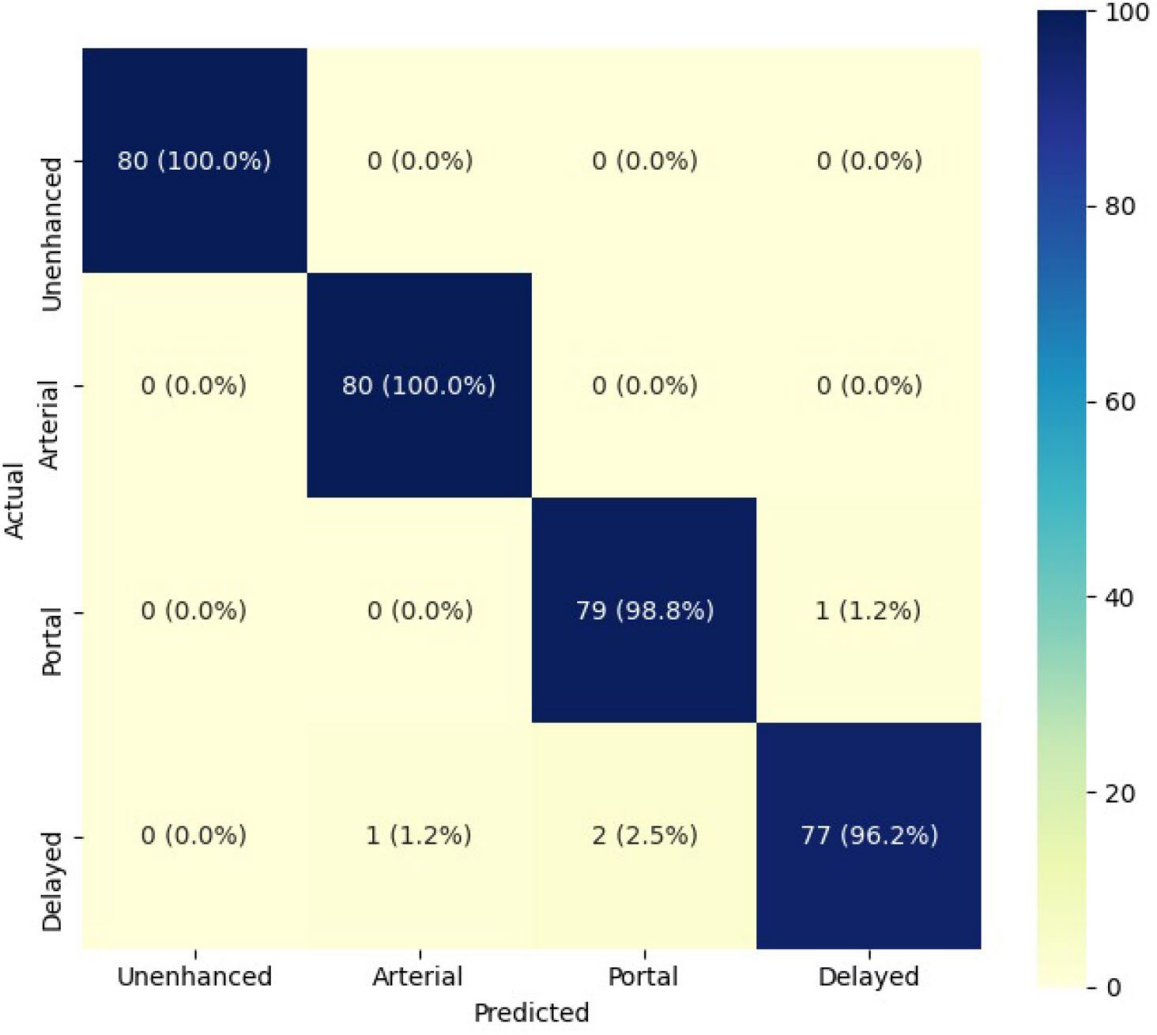
Confusion matrix heat map of the testing set volumes.

When combining each slice prediction into a single volume prediction, the accuracy rose to 98%. Increases were also observed for the other evaluation metrics, as shown in Table 4. Across the four phases, the F1-score and AUC metrics show a decrease in performance according to the order of occurrence of the phases. Particularly, the unenhanced has the best values while the delayed has the lowest ones. It is possible to observe in Fig. 8 that most mistakes in classification are in the portal and delayed phases.

**Table 4:** Performance metrics in the testing set for the volumes evaluation using the best model found by the Hyperparameter Tuning.

	Precision	Recall	F1-score	AUC	N
Unenhanced	1.00	1.00	1.00	1.00	80
Arterial	0.99	1.00	0.99	1.00	80
Portal	0.97	0.99	0.98	1.00	80
Delayed	0.99	0.96	0.97	0.99	80
Average	0.99	0.99	0.99	0.99	–

The accuracy of the exams evaluation was 100%, which means that all phases in all exams were correctly classified.

### Cross validation results

For a better evaluation of our model, we added a cross validation step using four folds. Despite the decreasing performance shown in Tables 5 and 6 , the general accuracy for slices and volumes was above 92%. The accuracy for the exams was 96.5 ± 3.93%.

**Table 5:** Performance metrics in the testing set for the slices evaluation using cross validation. The values are mean±standard deviation.

	Precision	Recall	F1-score	AUC	N
Unenhanced	0.98 ± 0.02	0.99 ± 0.01	0.99 ± 0.01	1 ± 0.00	10,839.25 ± 126.79
Arterial	0.97 ± 0.01	0.95 ± 0.04	0.96 ± 0.02	0.99 ± 0.01	10,972.75 ± 148.56
Portal	0.87 ± 0.06	0.87 ± 0.04	0.87 ± 0.02	0.98 ± 0.01	11,770.0 ± 129.03
Delayed	0.89 ± 0.03	0.88 ± 0.05	0.88 ± 0.01	0.98 ± 0.00	10,926.25 ± 174.68
Average	0.93 ± 0.01	0.92 ± 0.01	0.93 ± 0.01	0.99 ± 0.00	–

**Table 6:** Performance metrics in the testing set for the volumes evaluation using cross validation.

	Precision	Recall	F1-score	AUC	N
Unenhanced	0.99 ± 0.01	1.00 ± 0.00	0.99 ± 0.01	1.00 ± 0.00	80
Arterial	0.99 ± 0.01	0.97 ± 0.04	0.98 ± 0.02	0.99 ± 0.01	80
Portal	0.93 ± 0.05	0.97 ± 0.02	0.95 ± 0.03	0.99 ± 0.01	80
Delayed	0.97 ± 0.02	0.94 ± 0.02	0.96 ± 0.02	0.99 ± 0.00	80
Average	0.97 ± 0.02	0.97 ± 0.02	0.97 ± 0.02	0.99 ± 0.00	–

The values are mean ± standard deviation.

Overall, all metrics were higher in the volumes evaluation than in the slices one. For both slices and volumes, the highest metrics were achieved in the unenhanced and arterial phases.

Given that the exams used in this experiment are from four different CT machine manufacturers, we evaluated the accuracy of the exams for each manufacturer. The results show that there was not a significant difference among them and the accuracy is above 95% for all machines.

In addition, exams from patients with chronic liver disease (n = 65) have an accuracy of 96.5 ± 3.4%, while the exams from healthy patients (n = 15) have 96.7 ± 6.7%.

## Discussion

Herein, the CNN model developed to predict, identify and differentiate contrast-enhanced phases in liver computer tomography presented results with high accuracy.

Besides the accuracy achieved using each single slice as input being slightly higher than the one achieved by Dercle et al.^18^, who used a random forest classifier for predicting optimal-portal phase (85% in our analysis compared to 84% in theirs), the average accuracy of our algorithm rose to 98% in the volumes evaluation. In addition, our algorithm’s performance rose up to 99% when considering other evaluation metrics such as the AUC (0.997 ± 0.002). Hence, this result further demonstrates the potential of our approach to be applied in the clinical setting. In terms of the delayed phase’s accuracy, our findings are also in unison with Dercle et al.^18^, since this phase concentrated most of prediction mistakes among all the evaluated phases.

Nonetheless, it should be noted that these findings may be compared with some caution, since the input, algorithm and output data differ among our study and the one by Dercle et al.^18^. For instance, we aimed to precisely identify only four phases of the exams (unenhanced, arterial, portal and delayed), since the CT evaluation of the liver is most commonly based on this four-phase protocol rather than a five-phase one that includes an optimal-portal category described by Dercle et al.^18^. Furthermore, we developed a fully-automated solution using only imaging data as input for our CNN algorithm, as opposed to Dercle et al.^18^, who used the mean intensities of the abdominal aorta and portal vein extracted from specialist annotated pixels.

The success of our approach is also evidenced by the performance of the exams evaluation, which, to the best of our knowledge, had not been previously tested by any research group. Furthermore, we included a hyperparameter tuning step, which has been shown to slightly improve the accuracy of AI algorithms, being fundamental for any cutting-edge pipeline^23^. In addition, we showed that there is no variability among CT machines in terms of algorithms’ accuracy. Furthermore, one of the advantages of our approach does not need to use metadata in order to be applied to real data sets.

**Figure 9:**
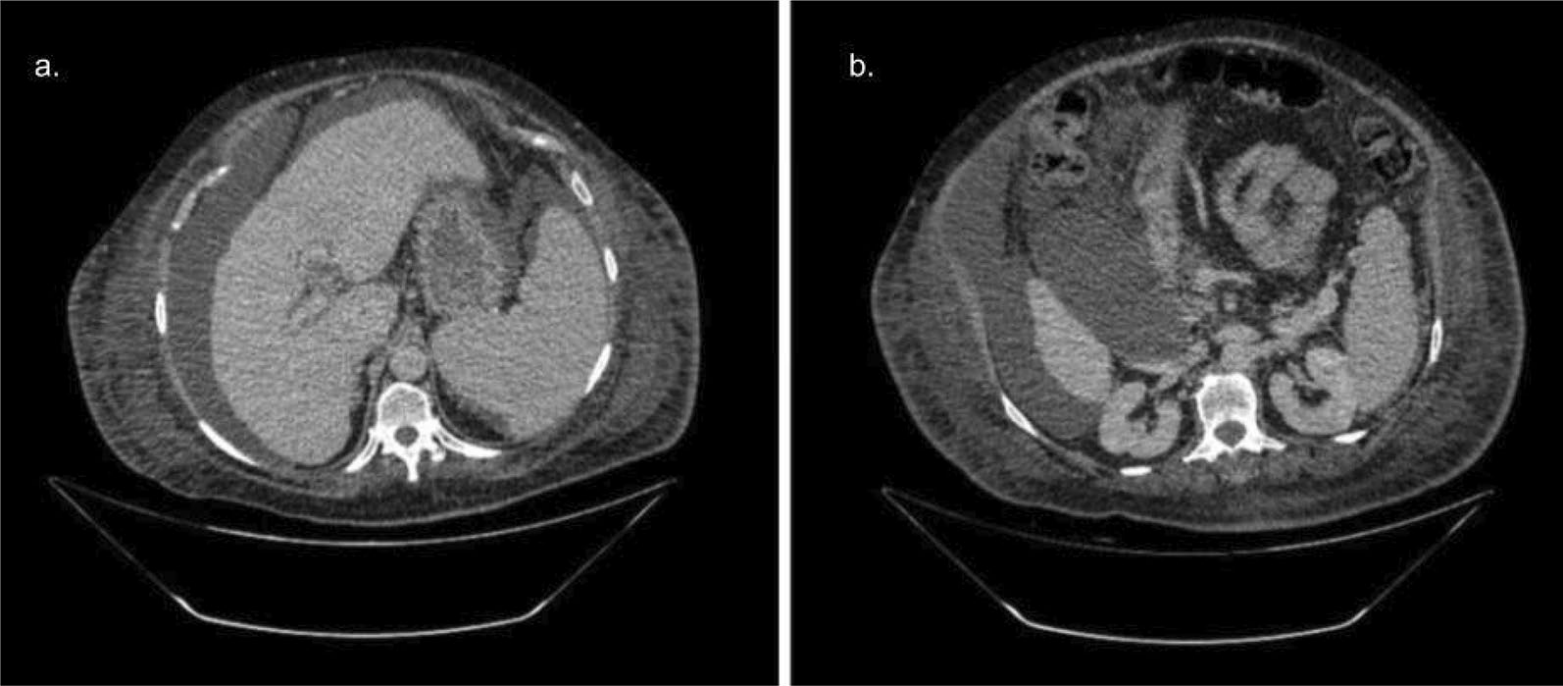
Two slices from the delayed phase incorrectly classified as portal phase. In general, in the delayed phase, we notice contrast being excreted in the urinary collecting system. In this case, despite being a delayed phase, no contrast was observed, probably due to low cardiac output or slow urinary function.

**Figure 10:**
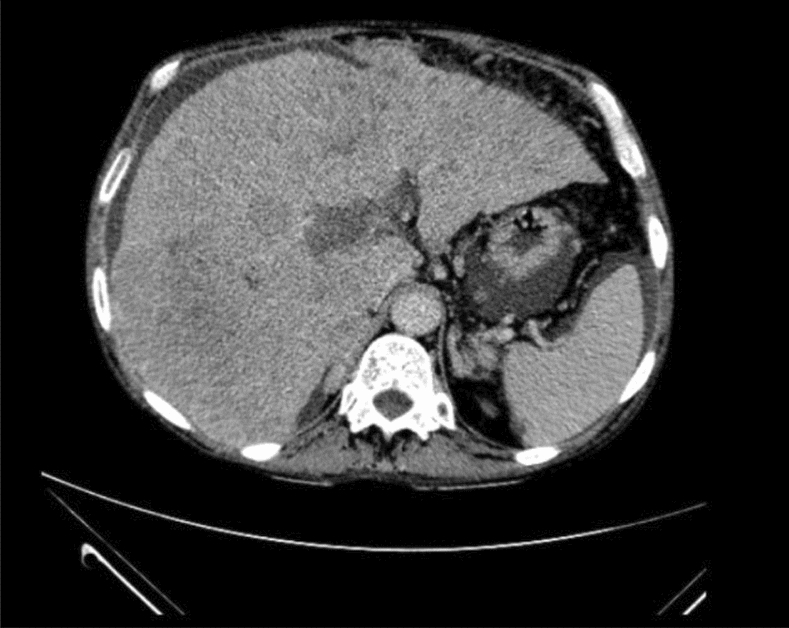
Example of a portal phase incorrectly classified as a delayed phase. Note that there is an infiltrative hepatocellular carcinoma with extensive portal vein thrombosis.

In the test set volume, the algorithm misclassified the exam series in four volumes. By analyzing each case individually, the reason behind the misclassification cannot be determined with certainty, but it is possible to infer potential confounding factors. In two cases, the algorithm classified a delayed phase as a portal as shown in Fig. 9. When analyzing the images, we observed that these correspond to patients whose delayed phase showed no contrast excreted in the collecting system. We deduced that, probably, the lack of contrast in the collecting system in these patients with possible slow cardiac output or slow renal excretion rate may be the confounding factor. In this kind of situation, a radiologist who only has access to this phase of the exam will also have difficulties in differentiating whether it is a delayed phase or a portal phase with poor quality. In the other two cases, the algorithm classified a portal phase as an delayed phase as shown in Fig. 10. There was extensive thrombosis of the trunk of the portal vein, causing the liver and the portal vein not to present significant enhancement, which may have been the confounding factor.

An important aspect to be taken into account in this study is the fact that it included healthy patients and mostly cirrhotic patients, including in very advanced stages. This composition of the patient profile shows that the algorithm performs well even in a population with a high prevalence of liver disease.

For instance, in a clinical environment, this kind of deep learning approach to identify contrast phase may also be useful for creating databases that facilitate radiologists’ daily routine and, ultimately, accelerate the diagnosis of liver diseases, identifying, selecting and organizing images within the DICOM viewer, based on post-contrast phases, saving one more step in the analysis. Besides, it could also allow the post-contrast images’ quality evaluation, bringing new possibilities for quality control tools.

Furthermore, a CT phase identifier can be used as a general, yet extremely useful strategy to organize and clean the input data for any algorithm that utilizes image information. As noted by Castaldo et al.^1^, these two issues are among the main limitations in the fields of radiomics and artificial intelligence. In this context, this AI solution can potentially aid in solving those issues and might be incorporated in other radiology-pipelines, improving the accuracy of virtually any CT abdominal algorithm that uses specific contrast phases as input.

These results show a promising application of a CNN-based phase identifier. However, it is necessary to evaluate it on a larger dataset of exams, especially with more diverse machines and image acquisition protocols to guarantee the maintenance of performance and subsequent practical implementation of this type of approach.

## Conclusion

Our study successfully demonstrates an approach to phase recognition of contrast-enhanced abdominal CT scans using a Convolutional Neural Network (CNN). Considering the high mean accuracy of our algorithm, our results can significantly aid in the proper and fundamental standardization and quality control of input data for liver segmentation and identification of HCC lesions. Further validation studies are necessary to check whether the algorithm’s accuracy remains the same for a diverse set of CT exams, especially for different patient profiles and institutions with potentially different infusion pumps and contrast timing protocols. Addressing this question will be fundamental to tailor AI solutions for a faster and more proper care of patients with liver diseases.

## Data Availability

The dataset generated during the current study is not publicly available due to institutional privacy policies but is available from the corresponding author on reasonable request.
